# Intrapersonal and interpersonal predictors of suicidal ideation and non-suicidal self-injury in a 12-month EMA follow-up

**DOI:** 10.1192/j.eurpsy.2026.10300

**Published:** 2026-07-31

**Authors:** A. Alacreu-Crespo, A. Porras-Segovia, E. Baca-García

**Affiliations:** 1 Psychology and Sociology, University de Zaragoza, Teruel; 2 Health Research Institute, Fundación Jiménez Díaz, Madrid, Spain

## Abstract

**Introduction:**

Suicide and non-suicidal self-injury (NSSI) remain critical public health challenges. Traditional clinical assessments are often limited by recall bias and a failure to capture the dynamic fluctuations of suicidal thoughts and behaviors. Ecological Momentary Assessment (EMA) has emerged as a solution, though most research is limited by short follow-up periods that fail to capture long-term clinical trajectories.

**Objective:**

Investigate the **real-time associations between suicidal ideation (SI) and NSSI** using long-term smartphone monitoring

**Methods:**

This prospective study utilized a smartphone-based EMA to monitor high-risk psychiatric outpatients (N≈100) over an extended period of 6 to 12 months. Participants received daily prompts with 2–4 randomized questions assessing suicidal ideation (SI), desire to self-harm (DSH), negative affect, and interpersonal experiences. Mixed-effects models were used to analyze within-person variability and temporal trends.

**Results:**

SI and NSSI are significantly associated with negative affect and interpersonal difficulties. High within-person variability was observed, suggesting that risk is a fluctuating state rather than a stable trait. Notably, the extended follow-up allowed for the identification of temporal slopes; for instance, while restlessness and sadness decreased over time in NSSI patients, passive SI and anxiety showed increasing trends—nuances often missed in typical 2–4 week studies.

**Conclusions:**

Long-term EMA is a feasible, less invasive methodology that provides a superior understanding of suicidal behavior. The primary advantages include the drastic reduction of recall bias and the unique ability to capture proximal, real-time predictors over a clinically meaningful duration. This longitudinal perspective is vital for transitioning from simple monitoring to actionable, personalized mHealth interventions.

**Disclosure of Interest:**

None Declared

